# Integrated Omics Insights into Dapagliflozin Effects in Sepsis-Induced Cardiomyopathy

**DOI:** 10.3390/biom15020286

**Published:** 2025-02-14

**Authors:** Weiwei Lai, Li Liu, Shuhang Wang, Yancun Liu, Yanfen Chai

**Affiliations:** Department of Emergency Medicine, Tianjin Medical University General Hospital, Tianjin 300052, China

**Keywords:** sepsis-induced cardiomyopathy, dapagliflozin, transcriptomics, metabolomics, cardioprotective mechanisms

## Abstract

Background: Sepsis-induced cardiomyopathy (SIC) is a life-threatening cardiac complication of sepsis with limited therapeutic options. Dapagliflozin, a sodium-glucose cotransporter 2 (SGLT2) inhibitor, has demonstrated cardioprotective effects in heart failure, but its role in mitigating sepsis-related cardiac dysfunction remains unclear. Methods: A retrospective cohort analysis was conducted to assess the impact of pre-hospital dapagliflozin use on major adverse cardiovascular events (MACEs) and survival in patients with SIC. Additionally, a murine SIC model was established using cecal ligation and puncture (CLP) to evaluate the effects of dapagliflozin on cardiac function, histopathology, and biomarkers of myocardial injury. Transcriptomic and metabolomic profiling, combined with multi-omics integration, was employed to elucidate the molecular mechanisms underlying dapagliflozin’s cardioprotective effects. Results: In the clinical cohort, pre-hospital dapagliflozin use was associated with a significant reduction in the risk of MACE and improved survival outcomes. In the murine SIC model, dapagliflozin restored cardiac function, reduced biomarkers of myocardial injury, and alleviated histological damage. Multi-omics analysis revealed that dapagliflozin modulates inflammatory responses, enhances autophagy, and regulates metabolic pathways such as AMPK signaling and lipid metabolism. Key regulatory genes and metabolites were identified, providing mechanistic insights into the underlying actions of dapagliflozin. Conclusions: Dapagliflozin significantly improves cardiac outcomes in sepsis-induced cardiomyopathy through the multi-level regulation of inflammation, energy metabolism, and cellular survival pathways. These findings establish dapagliflozin as a promising therapeutic strategy for SIC, offering translational insights into the treatment of sepsis-induced cardiac dysfunction.

## 1. Introduction

Sepsis-induced cardiomyopathy (SIC) is a frequent and severe complication of sepsis [1] characterized by impaired cardiac function and a high mortality rate [2,3]. Despite the potential for early intervention to reverse the condition, untreated SIC rapidly progresses, compromising the heart’s ability to pump blood, exacerbating tissue ischemia, and ultimately leading to multi-organ dysfunction or failure [4,5]. Effective management of SIC is, therefore, critical for improving patient survival rates and reducing the burden of sepsis-related complications.

Dapagliflozin, initially developed for the management of type 2 diabetes, has emerged as an effective therapy in heart failure (HF) over recent years [6,7]. Landmark clinical trials, such as DAPA-HF, have demonstrated that dapagliflozin significantly reduces cardiovascular mortality and hospitalizations in heart failure patients while also improving quality of life, irrespective of diabetes status [8,9,10]. Based on robust clinical evidence, sodium-glucose cotransporter 2 (SGLT2) inhibitors, including dapagliflozin, are now considered essential components of heart failure treatment guidelines [11,12]. Given dapagliflozin’s established role in heart failure management, its potential therapeutic benefit in sepsis-induced cardiomyopathy has generated significant interest.

Studies have shown that septic patients with SIC are at a significantly higher risk of major adverse cardiovascular events (MACEs) compared to those without SIC [13]. This elevated risk underscores the shared underlying pathophysiological mechanisms between SIC and heart failure, such as metabolic dysfunction, impaired energy utilization, and oxidative stress [14,15]. In heart failure, dapagliflozin has demonstrated robust cardioprotective effects by addressing these dysfunctions—improving metabolic pathways, reducing oxidative stress, and restoring myocardial energy homeostasis [16,17]. However, the specific effects of dapagliflozin in SIC remain underexplored [18,19], particularly with regard to its potential to mitigate sepsis-induced myocardial injury, similar to its role in heart failure management [20].

To address this gap in knowledge, our study aims to evaluate the efficacy of dapagliflozin in sepsis-induced cardiomyopathy [11]. We hypothesized that dapagliflozin may offer cardioprotective benefits by maintaining myocardial homeostasis, reducing cardiac damage, and improving survival outcomes. Clinical analysis confirmed that dapagliflozin significantly reduced major adverse cardiovascular events (MACEs) and improved survival in patients with sepsis-induced cardiomyopathy, suggesting its potential as a novel therapeutic strategy. To further investigate, we explored the cardioprotective mechanisms of dapagliflozin in a septic animal model. The results demonstrated that dapagliflozin pre-treatment significantly reduced myocardial injury, preserved cardiac function, and alleviated pathological stress. These findings collectively support the therapeutic potential of dapagliflozin in sepsis-induced cardiomyopathy and provide a compelling rationale for its broader clinical application in cardiovascular protection.

## 2. Methods

### 2.1. Patient Subjects

This study conducted a retrospective cohort analysis of patients with sepsis-induced cardiomyopathy treated at our hospital from September 2021 to September 2023. Relevant diagnostic and treatment information was collected using the hospital’s electronic medical record (EMR) system. All methods were confirmed to have been performed in accordance with relevant guidelines and regulations.

Patients meeting the inclusion criteria were (1) diagnosed with sepsis according to Sepsis 3.0 criteria; (2) aged 18 years or older; (3) presenting with troponin I (TnI) levels exceeding 0.4 ng/mL (hospital reference range: 0–0.4 ng/mL) and/or a left ventricular ejection fraction (LVEF) below 50% on echocardiography based on the first available results after admission; and (4) having complete clinical records. Exclusion criteria were (1) a history of chronic liver or kidney failure, heart failure, cardiomyopathy, valvular disease, myocardial infarction, ischemic stroke, autoimmune disorders, or malignancy; (2) age under 18; and (3) incomplete medical records.

Participants were categorized into two groups based on pre-hospital dapagliflozin use: (1) Dapagliflozin Use Group: Patients who had consistently taken dapagliflozin for at least seven consecutive days prior to admission. Consistent use was defined as a daily dose of dapagliflozin (10 mg/day) verified through EMR records, including pharmacy refill data, and supplemented by patient self-reports when available. (2) Non-Dapagliflozin Use Group: Patients who had not used dapagliflozin before admission. Patients who initiated dapagliflozin after admission were excluded from the study to eliminate potential confounding effects. The primary clinical outcome was defined as the incidence of major adverse cardiovascular events (MACEs) within one year of admission, including myocardial infarction, ischemic stroke, heart failure, or cardiovascular death [13,21].

### 2.2. Animal Model Construction

C57BL/6J male *mice* (8–10 weeks old, weighing between 22–25 g) were randomly assigned to one of three groups: Sham group (sham surgery + saline), CLP group (CLP surgery + saline), and CD group (CLP surgery + dapagliflozin 10 mg/kg), with 7 *mice* in each group. The sample size was determined to meet the requirements for animal studies (n ≥ 3) and ensure the reliability of the results. Dapagliflozin (purchased from Aladdin, catalog number D101258-100 mg, Aladdin, Shanghai, China) was administered via gastric gavage for one week. *Mice* in the Sham and CLP groups received an equivalent volume of saline as a control. The sepsis-induced cardiomyopathy (SIC) model was established according to previous studies and adapted to the laboratory conditions. After anesthetizing the *mice* with isoflurane (3% for induction, 2% for maintenance), a 1.5 cm midline abdominal incision was made to expose the cecum. The left side of the abdomen was shaved and disinfected before surgery. A 3-0 silk suture was used to ligate approximately 50% of the distal cecum (measured as a percentage of the total cecal length). Following ligation, an 18G needle was used to puncture the center of the distal cecum, causing a slight extrusion of fecal matter to induce infection. The cecum was then returned to its original position, and the abdominal cavity was sutured [22,23,24]. On postoperative day 1, *mice* were given subcutaneous injections of saline (50 mL/kg) for resuscitation, without the use of antibiotics, to evaluate systemic inflammatory response. Sham group *mice* underwent the same procedures except for cecal ligation and puncture. According to the guidelines of the National Institutes of Health, all animal experiments were approved by the Animal Ethics Committee of Tianjin Medical University General Hospital (Ethical License No: IRB2022-KY-403).

### 2.3. Echocardiography

Twenty-four hours post-CLP surgery, *mice* were anesthetized with 1% isoflurane to ensure sufficient depth of anesthesia, minimizing stress and movement (*n* = 4). Cardiac function was assessed at this standardized timepoint using a Vevo 3100 small animal ultrasound system with a high-frequency probe (40 MHz)(manufacturer: Fujifilm VisualSonics, Toronto, Canada). Echocardiographic evaluations focused on key parameters, including left ventricular ejection fraction (LVEF), fractional shortening (FS), left ventricular end-diastolic volume (LVEDV), and end-systolic volume (LVESV) [25]. All echocardiographic evaluations were conducted by a single experienced operator under standardized conditions to minimize variability. Imaging parameters were optimized for mouse cardiac evaluation, and measurements were averaged across three consecutive cardiac cycles to ensure accuracy and consistency.

### 2.4. Biochemical Examination and Enzyme-Linked Immunosorbent Assay

Following echocardiography, blood samples were collected via cardiac puncture under anesthesia (n = 4). The collected blood was allowed to clot at room temperature before serum separation. The samples were centrifuged, and the serum supernatant was collected for analysis. Serum markers related to myocardial injury and function [26], such as CK-MB and LDH, were measured using a biochemical analyzer(URIT Medical Electronic Co., Ltd., Guilin, China) following the manufacturer’s protocols. Simultaneously, myocardial tissue samples were harvested immediately after blood collection, homogenized, and centrifuged to obtain tissue supernatants. The concentrations of cTnT and BNP in the myocardial tissue supernatants were measured using ELISA kits (ABclonal, Wuhan, China) following the manufacturer’s instructions.

### 2.5. Histologic Analysis

After blood collection, heart tissues from each group (n = 4) were immediately excised and rinsed with cold PBS to remove blood and impurities. Tissues were fixed in 4% paraformaldehyde, processed, and embedded in paraffin. Sections were stained with hematoxylin and eosin (H&E) to assess general histological structures and Masson’s trichrome to evaluate fibrosis(Beyotime Biotechnology, Shanghai, China). Myocardial tissue damage was scored on a scale of 0 to 3 as follows: 0 (normal): intact myocardial structure with well-arranged fibers, no nuclear swelling or pyknosis, and no congestion, edema, or inflammation; 1 (moderate): clear structure, few cells with nuclear swelling or pyknosis, mild congestion, edema, and inflammation; 2 (severe): disorganized structure, significant nuclear swelling or pyknosis, marked congestion, edema, and inflammation; 3 (critical): disrupted structure, ruptured fibers, extensive nuclear swelling or pyknosis, and diffuse congestion, edema, and inflammation. For Masson’s trichrome staining, fibrosis quantification was performed using ImageJ_v1.8.0 to measure the percentage of collagen content in the myocardial tissue. Morphological assessments were performed systematically, and all samples were analyzed by an experienced pathologist to ensure reliability and consistency in interpretation.

### 2.6. Transcriptomics

After blood collection, a portion of the excised heart tissue was immediately snap-frozen in liquid nitrogen and stored at −80 °C for transcriptomic analysis (n = 4). Total RNA was extracted from heart tissues, and its quality and purity were assessed using a Bioanalyzer(Agilent 2100, Agilent Technologies, Santa Clara, CA, USA). Only high-quality RNA samples (RNA integrity number [RIN] > 7) were used for library preparation and sequencing. During preprocessing, low-quality reads and adapter sequences were filtered out to ensure robust data. Gene expression levels were quantified, and differential expression analysis was conducted using DESeq2. The thresholds for differential expression were set at |log2(FoldChange)| ≥ 1 and an adjusted *p*-value ≤ 0.05.

### 2.7. Untargeted Metabolomics Studies

Heart tissues from each group (n = 7) were collected 24 h post-CLP surgery, homogenized, and centrifuged to extract supernatants for untargeted metabolomics analysis using LC-MS(Thermo Fisher Scientific, Q Exactive, Waltham, MA, USA). Quality control procedures ensured reliable quantification and annotation of metabolites, which were mapped to metabolic pathways using the KEGG database to explore their biological roles. Principal component analysis (PCA) was conducted to identify variance trends, followed by Orthogonal Partial Least Squares Discriminant Analysis (OPLS-DA) to distinguish metabolic characteristics among the Sham, CLP, and CD groups. Differential metabolites were identified based on the criteria of VIP > 1.0, FC > 1.2 or < 0.833, and *p*-value < 0.05. Hierarchical clustering and correlation analyses were further performed to investigate relationships among samples and metabolites. Receiver Operating Characteristic (ROC) curve analysis was employed to evaluate the diagnostic potential of key metabolites by calculating the area under the curve (AUC) for each metabolite, assessing their ability to differentiate between experimental groups. Additionally, random forest analysis was conducted using the Mean Decrease Accuracy metric to rank metabolites by their importance in the classification model, providing insights into their roles in distinguishing metabolic profiles across groups.

### 2.8. Metabonomics–Transcriptomics Combination Analysis

Differential metabolites (DEMs) and genes (DEGs) were identified for joint analysis. DEMs were annotated using the KEGG database to map their functions and related metabolic pathways. Subsequently, DEMs and corresponding DEGs were integrated into metabolic pathways to explore interactions within the biological network. This pathway analysis revealed significant changes across experimental groups, offering insights into the molecular mechanisms of dapagliflozin’s cardioprotective effects in sepsis-induced cardiomyopathy.

### 2.9. Statistical Analysis

Statistical analyses were conducted using SPSS 27.0 and R 4.3.0. Continuous variables were reported as mean ± standard deviation or median with interquartile range, while categorical variables were expressed as frequencies and percentages. Significant variables identified during analysis were adjusted for confounding factors as needed. Graphs and tables were created using GraphPad Prism 10 and R Studio.

## 3. Results

### 3.1. Dapagliflozin Enhances Cardiovascular Protection in Patients with SIC

**Figure 1:**
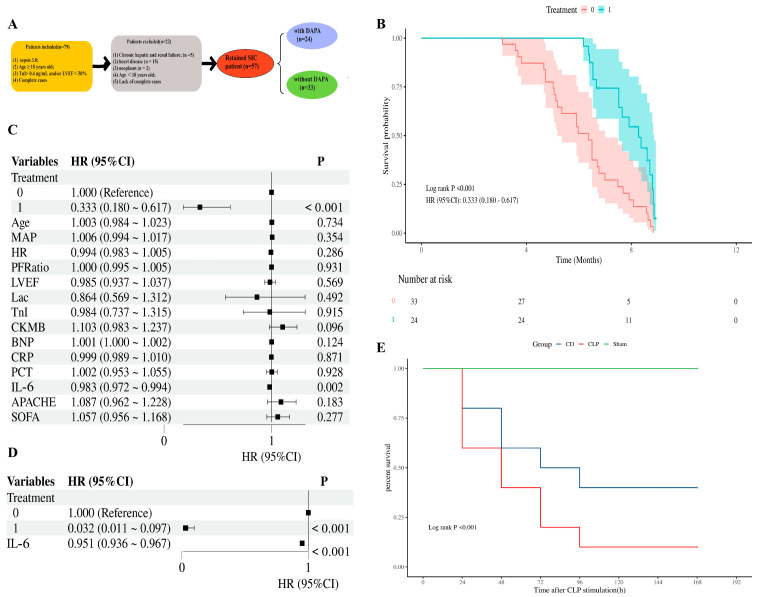
SIC patient selection and survival analysis with COX model. (**A**) Flowchart of SIC patient selection. From 79 patients, 22 were excluded (chronic liver/kidney failure, heart disease, coagulopathy, age limits, incomplete data), leaving 57 patients: 24 with DAPA and 33 without; (**B**) Kaplan–Meier survival curves comparing the two treatment groups (treatment 1: pre-hospital use of DAPA vs. treatment 0: no pre-hospital use of DAPA). The log-rank test is shown, with HR (95% CI) values indicated; (**C**,**D**) COX proportional hazards regression models were used to evaluate the impact of treatment and other variables on survival outcomes: (**C**) univariate and (**D**) multivariate analyses. Hazard ratios (HRs), 95% confidence intervals (CIs), and *p*-values are reported for key clinical parameters. HR: hazard ratio, CI: confidence interval; (**E**) dapagliflozin intervention on 7-day survival in *mice* with sepsis (*n* = 10).

In this study, 57 patients diagnosed with sepsis-induced cardiomyopathy were selected based on predefined exclusion criteria (Figure 1A). Of these, 24 patients had used dapagliflozin (DAPA) prior to hospital admission, while 33 had not. Baseline comparisons between the two groups revealed statistically significant differences in SOFA score, CKMB, BNP, and APACHE score (*p* < 0.05, Table 1).

Our survival analysis, utilizing Kaplan–Meier curves (Figure 1B), revealed a clear distinction between the two groups: patients with prior dapagliflozin (DAPA) use demonstrated a significantly higher survival probability compared to those who had not used DAPA. The log-rank test confirmed the statistical significance of this difference (*p* < 0.001), highlighting a substantial survival benefit associated with pre-hospital DAPA administration. Univariate Cox regression analysis indicated that DAPA use was significantly associated with a reduced risk of mortality, with a hazard ratio (HR) of 0.333 (95% CI: 0.180–0.617), *p* < 0.001(Figure 1C). These findings suggest that DAPA may offer significant protection in terms of clinical outcomes for patients with SIC. However, it is important to note that univariate analysis evaluates the relationship between individual variables and the outcome without adjusting for potential confounders. To more rigorously evaluate the independent effect of DAPA, we performed multivariate Cox regression analysis, adjusting for potential confounding factors such as sepsis severity (measured by APACHE II and SOFA scores), inflammatory biomarkers (e.g., IL-6), and other relevant clinical variables. In the multivariate model, the protective effect of DAPA was even more pronounced, with an HR of 0.032 (95% CI: 0.011–0.097), *p* < 0.001. This further strengthens the hypothesis that DAPA plays a robust role in reducing the risk of adverse outcomes in SIC patients(Figure 1D).

### 3.2. Dapagliflozin Alleviates Myocardial Dysfunction in SIC mice

**Figure 2:**
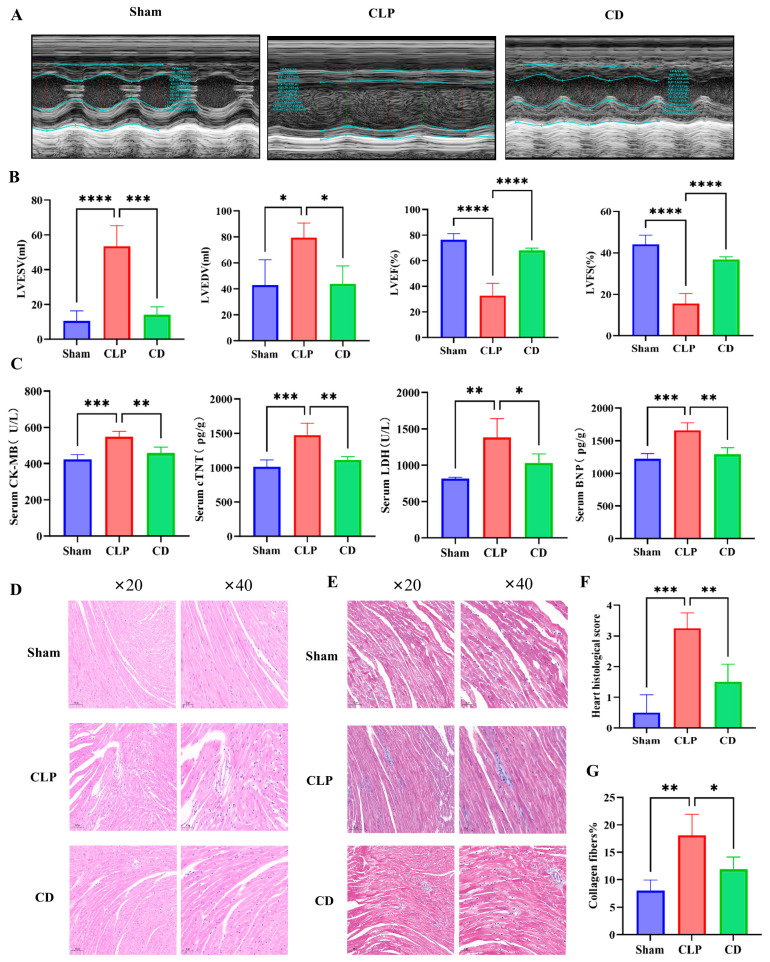
Dapagliflozin alleviates myocardial dysfunction in SIC *mice*. (**A**) Representative images of M-mode echocardiography (*n* = 4); (**B**) quantitative analysis of LVESV, LVEDV, LVEF, and LVFS in each group of *mice* (*n* = 4); (**C**) measurements of myocardial injury markers: cTnT and BNP levels were assessed from myocardial tissue supernatants, while CK-MB and LDH levels were measured from serum samples (*n* = 4); (**D**) representative images of HE staining; (**E**) representative images of MASSON staining; (**F**) histopathological score of the heart (*n* = 4); (**G**) quantitative analysis of collagen deposition: the percentage of collagen-positive area was calculated as the ratio of blue-stained regions to the total tissue area, reflecting the degree of fibrosis. * Indicates a statistically significant *p* < 0.05. ** Indicates a statistically significant *p* < 0.01; *** Indicates a statistically significant *p* < 0.001; **** indicates a statistically significant *p* < 0.0001.

Kaplan–Meier survival analysis of *mice* demonstrated significant differences in survival rates among the three groups (*p* < 0.001). The survival rate in the CLP group was significantly reduced, consistent with previous studies describing moderate to severe sepsis models, thereby validating the effectiveness of the CLP model in simulating severe sepsis. In contrast, the CD group showed an improved survival rate, indicating that dapagliflozin mitigated the lethality associated with CLP. To further investigate the physiological basis underlying these survival differences, cardiac function was evaluated (Figure 1E). Cardiac function results showed that the CLP group experienced a significant decrease in LVEF and LVFS, along with an increase in LVESV and LVEDV, within 24 h post-induction compared to the Sham group. In contrast, the dapagliflozin pre-treatment group (CD group) demonstrated notable improvements in LVEF and LVFS and reductions in LVESV and LVEDV compared to the CLP group. These findings suggest that dapagliflozin pre-treatment effectively mitigated cardiac dysfunction in SIC *mice* (Figure 2A,B).

Additionally, biomarkers of myocardial injury, including BNP, cTnT, CK-MB, and LDH, were evaluated. These biomarkers were significantly elevated in the CLP group compared to the Sham group, indicating substantial myocardial injury induced by CLP. In the CD group, biomarker levels were notably reduced, highlighting the protective effects of dapagliflozin in alleviating myocardial injury (Figure 2C-G).

The histological analysis of cardiac tissue (Figure 2D,F) further supported the above observations. Hematoxylin and eosin (HE) staining showed normal myocardial structure in the Sham group, with well-organized myocardial cells and no pathological changes. In contrast, the CLP group exhibited significant myocardial damage, including disorganized and ruptured myocardial fibers, myocytolysis, nuclear fragmentation, capillary dilation, infiltration of numerous inflammatory cells, and features of apoptosis such as chromatin condensation, cellular shrinkage, and the presence of apoptotic bodies. In the CD group, myocardial damage was significantly reduced, and myocardial structure appeared closer to that of the Sham group, with only mild edema and minimal inflammatory cell infiltration. Although Masson’s trichrome staining did not reveal distinct infarction areas or collagen deposition, considerable interstitial fibrosis was still observed in the CLP group (Figure 2E,G). Overall, dapagliflozin pre-treatment significantly improved myocardial dysfunction and reduced tissue damage in SIC *mice*.

### 3.3. Transcriptomics Reveal Dapagliflozin’s Regulatory Effect on Gene Expression in SIC Mice

To further investigate the underlying mechanisms of dapagliflozin at the genetic level, transcriptomic analysis was performed on each group of *mice*. Strict quality control ensured the reliability and accuracy of the data. Quantitative and distribution analyses of gene expression demonstrated high consistency among samples in each group (Sham, CLP, CD), with uniformly distributed outliers. Correlation analysis between samples indicated that the Pearson correlation coefficient squared (R^2^) for most biological replicates was greater than 0.9, suggesting good reproducibility within groups. Principal component analysis (PCA) showed that the gene expression profile of the CD group was more similar to the Sham group, while it significantly differed from the CLP group. This suggests that dapagliflozin may effectively reverse the gene expression changes induced by SIC.

**Figure 3:**
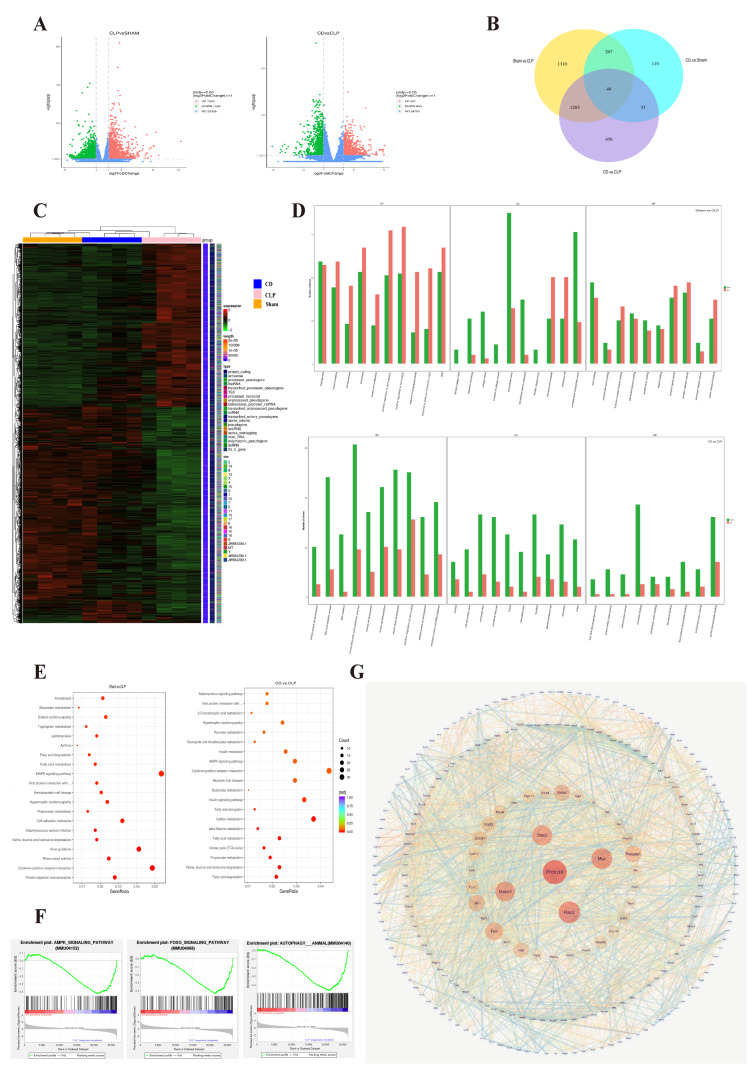
Transcriptomic analysis of dapagliflozin’s regulatory effects on gene expression in SIC mice (*n* = 4). (**A**) Volcano plot of differential gene expression: The x-axis shows log2FoldChange values, and the y-axis displays −log10padj or −log10pvalue. The blue dashed line indicates the differential gene screening threshold; (**B**) Venn diagram of differential genes: different colors represent various comparison groups; (**C**) Clustering heat map of differentially expressed genes; In the figure, the abscissa is the sample name, and the ordinate is the normalized value of the differential gene FPKM. The redder the color, the higher the expression level, and the greener the expression level, the lower the expression level. The chromosome to which each gene belongs, the length of the gene and the biological type of the gene are also added to the heat map; (**D**) GO enrichment analysis upward and downward histogram; In the figure, the abscissa is the GO Term, and the ordinate is the number of up-and down-regulated differential genes annotated on the GO Term; (**E**) KEGG enrichment distribution plot; In the figure, the abscissa is the ratio of the number of differential genes annotated to the KEGG pathway to the total number of differential genes, and the ordinate is the KEGG pathway; (**F**) GSEA enrichment analysis,Profile of the Running ES Score & Positions of GeneSet Members on the Rank Ordered List; (**G**) Protein interaction network diagram; Note: Each node in the figure represents a protein, and each connecting line represents the interaction between connected proteins.

Differential gene expression analysis (Figure 3A) further revealed that the CLP group, compared to the Sham group, significantly influenced the expression of numerous genes, with 1345 genes upregulated and 1563 genes downregulated, highlighting the substantial disruption caused by SIC. In comparison to the CLP group, the CD group had 967 genes upregulated and 853 genes downregulated, indicating that dapagliflozin significantly modulated the gene expression changes induced by SIC and suggesting an important impact on SIC. Compared to the Sham group, the CD group showed only 260 genes upregulated and 198 genes downregulated, indicating that dapagliflozin brought gene expression closer to normal levels in SIC *mice*.

Venn diagram analysis showed that 267 differentially expressed genes (DEGs) were shared between the comparisons of CLP vs. Sham and CD vs. Sham, indicating that dapagliflozin may mitigate the pathological changes induced by SIC by regulating these genes (Figure 3B). Heatmap analysis illustrated the gene expression trends across the groups and their similarities. Clustering analysis revealed that the gene expression profiles of the Sham and CD groups were more similar, while the CLP group remained distinct, further indicating that dapagliflozin was able to partially restore normal gene expression, particularly in metabolic, inflammatory, and immune-related pathways (Figure 3C).

To further explore the molecular mechanisms underlying these transcriptional changes, Gene Ontology (GO) and Kyoto Encyclopedia of Genes and Genomes (KEGG) enrichment analyses, as well as gene set enrichment analysis (GSEA) of hub genes, were conducted. GO enrichment analysis indicated that in the SIC model, biological processes related to inflammation, chemotaxis, and extracellular matrix remodeling were significantly altered, suggesting that SIC affects key biological processes relevant to its pathogenesis. Dapagliflozin significantly influenced biological processes related to cell migration, inflammation, and extracellular matrix remodeling, implying that it might alleviate cardiac damage in SIC by regulating these processes(Figure 3D).

According to KEGG enrichment analysis, pathways associated with metabolism, inflammation, immune response, infection, and cardiovascular disease were significantly enriched in the CLP group, indicating that SIC affects metabolic, immune, and cardiovascular functions through multiple pathways, which aligns with its complex pathogenesis. Dapagliflozin may exert its protective effects by modulating a variety of metabolic and signaling pathways, especially those involved in fatty acid metabolism, carbon metabolism, AMPK signaling, insulin resistance, and cytokine–cytokine receptor interactions (Figure 3E). These results demonstrate the role of dapagliflozin in regulating energy metabolism and balancing cellular responses, which contributes to the improvement of SIC pathologies. GSEA results further suggested that SIC leads to significant changes in metabolic processes (such as carbon metabolism, fatty acid degradation), autophagy, and insulin signaling pathways, while dapagliflozin pre-treatment could partially reverse these changes (Figure 3F).

Protein–protein interaction (PPI) network analysis (Figure 3G) identified key hub genes that occupy central positions in the network, including *Prdm10* (D = 113), *Rac2* (D = 102), *Myc* (D = 97), *Stat3* (D = 93), and *Mapk3* (D = 92). These genes are likely pivotal in the development of SIC and in mediating the effects of dapagliflozin treatment, particularly in the regulation of inflammatory responses, autophagy, and apoptosis, suggesting that they may serve as important targets for the therapeutic effects of dapagliflozin [27,28,29,30,31].

### 3.4. Metabolomics Reveal Dapagliflozin’s Regulatory Effect on Metabolic Imbalance

**Figure 4:**
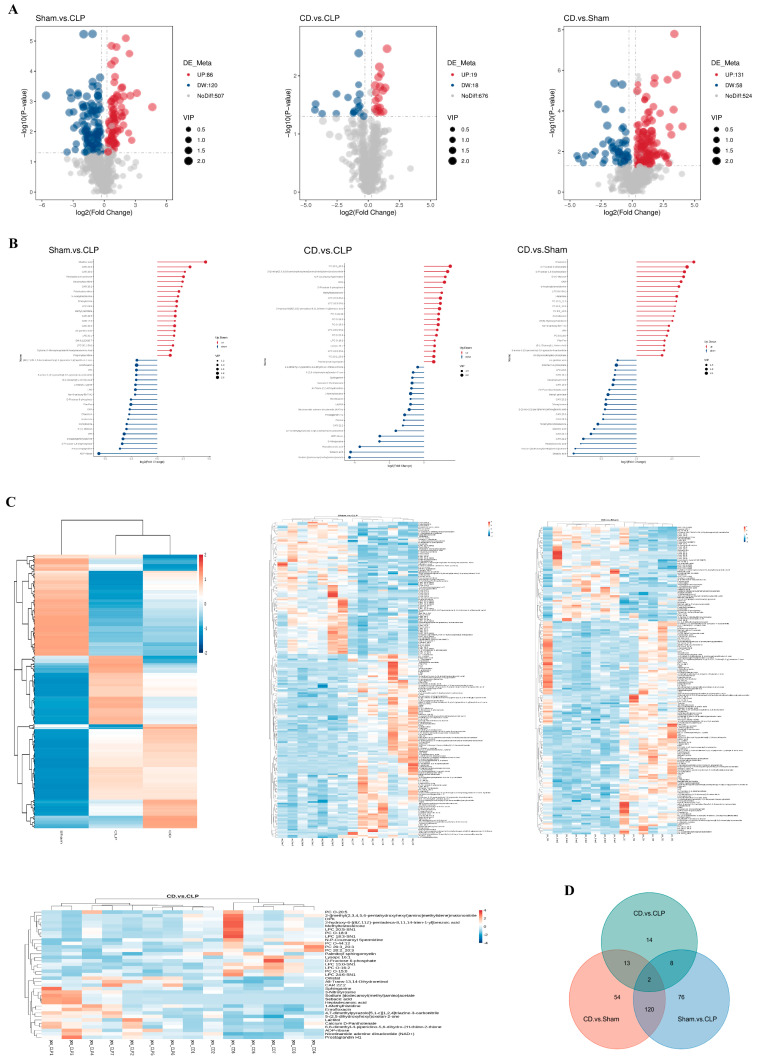
Impact of dapagliflozin on metabolic alterations (*n* = 7). (**A**) Volcano plot of differential gene expression: The x-axis represents log2FoldChange, and the y-axis is −log10padj or −log10pvalue. The blue dashed line shows the differential gene screening threshold; (**B**) Stick/bar plot of differential metabolites: The top 20 upregulated (red) and downregulated (blue) metabolites, sorted by log2(Fold Change), are displayed. Dot color indicates regulation direction (red: upregulated; blue: downregulated). Stick length corresponds to the magnitude of log2(Fold Change), and dot size represents VIP (Variable Importance in Projection) values; (**C**) Hierarchical clustering analysis of differential metabolites: Vertical dendrograms represent sample clustering, and horizontal dendrograms represent metabolite clustering. Shorter dendrogram branches indicate higher similarity. (**D**) Venn diagram of differential genes: various colors correspond to distinct comparison groups.

In metabolomics analysis, the Pearson correlation coefficient (R^2^) for each quality control (QC) sample was close to 1, indicating a strong correlation between QC samples and confirming the stability and reproducibility of the detection process. PCA and OPLS-DA models identified 713 metabolites in positive ion mode and 364 in negative ion mode. Volcano plots and stick plots visually presented the overall distribution of differentially expressed metabolites between the groups, revealing significantly upregulated or downregulated metabolites. A comparison of metabolite differences between experimental groups showed that the difference between the CD and CLP groups was relatively small, mainly involving lipid metabolism, energy metabolism, and cell membrane regulation, indicating that dapagliflozin selectively modulates certain metabolic processes during SIC treatment (Figure 4A,B).

**Figure 5:**
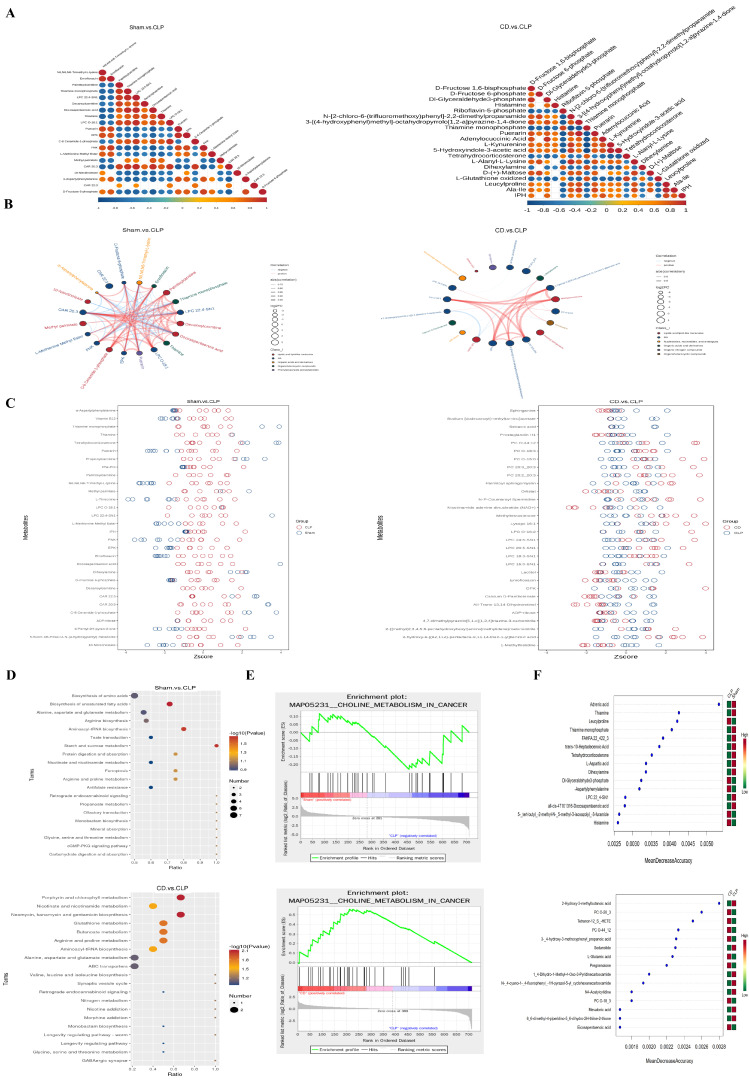
Comprehensive visualization of dapagliflozin’s effects on differential metabolites and pathway enrichment. (**A**) Correlation diagram of differential metabolites: Correlation ranges from 1 (complete positive, red) to −1 (complete negative, blue). Uncolored areas indicate *p*-value > 0.05. The top 20 metabolites are ordered by *p*-value; (**B**) chord chart of differential metabolites: Line thickness shows correlation strength; red lines denote positive correlations; and blue lines denote negative correlations. Only correlations with an absolute value > 0.7 are displayed; (**C**) Z-score plot: The x-axis is the Z-score value, and the y-axis lists differential metabolites. Each circle represents a sample. Only the top 30 metabolites (sorted by *p*-value) are shown; (**D**) KEGG enrichment bubble chart: The x-axis represents the ratio of differential metabolites in each pathway. Point size reflects the number of metabolites, and color indicates *p*-value significance, with smaller values showing greater statistical reliability; (**E**) GSEA enrichment analysis: depicts enrichment profiles for pathways affected by metabolites; (**F**) The x-axis represents the average decline (MeanDecreaseAccuracy) in model prediction accuracy when a specific feature (metabolite) is excluded. Higher values indicate greater importance in distinguishing between experimental groups. The heat map on the right shows the relative abundance of key metabolites across groups, with a color gradient from red (higher abundance) to green (lower abundance).

To better understand the impact of dapagliflozin on specific metabolites and its potential regulatory mechanisms, the relative quantification values of all differentially expressed metabolites were normalized to eliminate the differences in magnitude between metabolites, followed by clustering analysis of differentially expressed metabolites (Figure 4C). The results showed significant metabolic changes in the CLP group, reflecting metabolic disturbances caused by SIC. In the CD group, metabolite expression levels were closer to those in the Sham group, suggesting that dapagliflozin mitigated the metabolic disturbances induced by SIC and helped restore metabolic balance. Additionally, a correlation analysis of differentially expressed metabolites was conducted to explore the relationships between metabolites (Figure 5A–C). The top 20 differentially expressed metabolites, ranked by *p*-value, are presented to further elucidate the interactions and balance of metabolic processes in SIC following dapagliflozin treatment.

Based on KEGG enrichment results, the main biochemical pathways and signal transduction pathways involving differentially expressed metabolites were identified(Figure 5D). Compared to the Sham group, the metabolites in the CLP group were enriched in pathways related to metabolic disorders, such as Cushing’s syndrome and cortisol synthesis and secretion. In the comparison between the CD and CLP groups, differentially expressed metabolites were mainly enriched in pathways such as AMPK signaling and porphyrin and chlorophyll metabolism. The quantification of these metabolites was used for gene set enrichment analysis (GSEA) of KEGG terms (Figure 5E), which revealed that the activity of the “Choline Metabolism in Cancer” pathway was significantly reduced in the CLP group, while it was positively enriched in both the CD and Sham groups. This indicates that dapagliflozin partially reversed the inhibition of choline metabolism by SIC, highlighting the importance of choline metabolism in SIC.

Furthermore, ROC curve analysis highlighted the diagnostic and therapeutic relevance of dapagliflozin-associated metabolites in SIC. A total of 181 metabolites exhibited high diagnostic accuracy, with area under the curve (AUC) values exceeding 0.9 between the Sham and CLP groups. Additionally, 10 metabolites between the CD and CLP groups showed similarly high AUC values, reinforcing their potential for distinguishing between these groups. Key metabolites, such as Capryloylglycine, D-α-Hydroxyglutaric acid, 2-hydroxy-6-[(8Z,11Z)-pentadeca-8,11,14-trien-1-yl]benzoic acid, and Decanoylcarnitine (DPK), consistently displayed high AUC values, underscoring their potential as biomarkers for disease diagnosis and the therapeutic effects of dapagliflozin.

To further validate the role of these metabolites, random forest analysis was employed to rank differential metabolites based on their contribution to the classification model. Using the Mean Decrease Accuracy metric, several metabolites were identified as significant contributors to group discrimination (Figure 5F). In the CLP group, adrenic acid levels were significantly reduced, while histamine levels were notably increased. Conversely, the CD group exhibited significantly higher levels of tetranor-12 S-HETE and eicosapentaenoic acid (EPA) compared to the CLP group. These findings highlighted metabolic shifts between the groups and further supported the cardioprotective effects of dapagliflozin in SIC. The integration of ROC curve and random forest analyses revealed the importance of key metabolites in the therapeutic effects of dapagliflozin: Tetranor-12 S-HETE, a downstream product of arachidonic acid metabolism, plays a pivotal role in inflammation regulation. Its increased levels in the CD group suggest that dapagliflozin may alleviate systemic and cardiac inflammation in SIC by modulating the arachidonic acid pathway. Eicosapentaenoic acid (EPA), an omega-3 polyunsaturated fatty acid with well-documented anti-inflammatory and cardioprotective properties, was significantly elevated in the CD group. This increase supports its role in mitigating myocardial injury and improving cardiac function. These results emphasize that the identified metabolites not only serve as potential biomarkers for SIC diagnosis but also provide mechanistic insights into the metabolic reprogramming and anti-inflammatory effects of dapagliflozin. The combined use of ROC curve and random forest analyses strengthens the evidence for the role of metabolic regulation in the pathophysiology of SIC and dapagliflozin’s therapeutic benefits.

### 3.5. Integrated Analysis Reveals the Synergistic Regulation of Genes and Metabolites by Dapagliflozin

**Figure 6:**
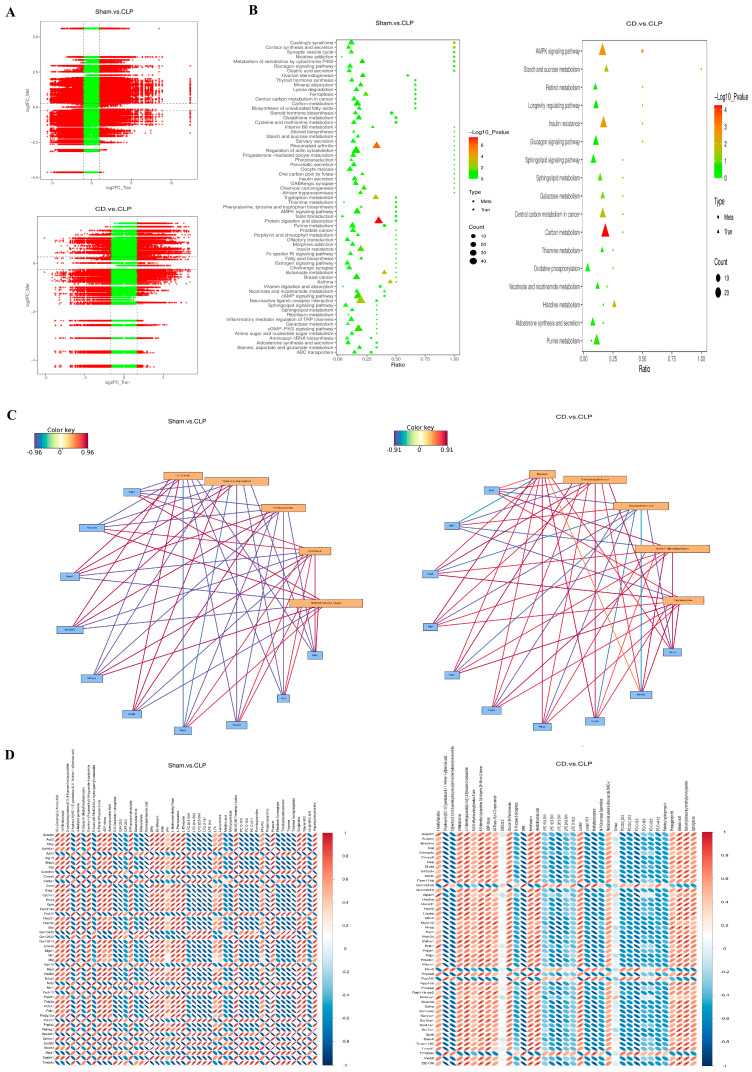
Integrated analysis of synergistic regulation of genes and metabolites by dapagliflozin. (**A**) Nine-image plot of differential metabolites and transcripts: The x-axis shows log2 fold change of transcripts (log2FC_Tran), and the y-axis shows log2 fold change of metabolites (log2FC_Met). Different groups are marked in red and green; (**B**) KEGG enrichment bubble chart: The x-axis is the ratio of metabolites or genes enriched in a pathway to the total annotated in the pathway. The y-axis lists KEGG pathways enriched by both metabolome and transcriptome. Dot size represents the number of enriched features, and color indicates *p*-value, with brighter colors signifying greater significance; (**C**) correlation network diagram: Yellow boxes represent metabolites, and blue boxes represent genes. Lines indicate correlations: red for positive and blue for negative. Darker colors correspond to higher correlation coefficients; (**D**) correlation heat map: Displays the relationship between differential metabolites and genes (positive ion mode). The y-axis represents metabolites, and the x-axis represents genes. Red indicates positive correlation; blue indicates negative correlation. The plot shows the top 500 metabolites and genes sorted by *p*-value.

By analyzing the differences in gene and metabolite expression between different experimental groups and calculating the correlation between all genes and metabolites using the Pearson correlation method, we observed significant differences in gene and metabolite expression between the CLP and Sham groups. In contrast, the changes in gene expression between the CD and Sham groups were minimal, suggesting that dapagliflozin partially alleviated the gene expression disorder induced by SIC. Additionally, the number of upregulated and downregulated metabolites in the CD group was roughly equal, indicating that dapagliflozin selectively modulated specific metabolic pathways crucial for cardioprotection rather than restoring all metabolic abnormalities indiscriminately (Figure 6A).

The correlation network between metabolites and genes (Figure 6C,D) provides a visual representation of the relationships between selected top differentially expressed metabolites and genes. Among these, Pdk1 and Retreg1 consistently demonstrated key correlations, underscoring their central roles in the metabolic and inflammatory processes associated with SIC. Between the CD and CLP groups, Pdk1 exhibited a negative correlation with eicosapentaenoic acid (EPA), suggesting that dapagliflozin alleviates inflammation-driven metabolic stress by modulating Pdk1, consistent with EPA’s anti-inflammatory properties. A positive correlation with Capryloylglycine further highlights Pdk1’s role in enhancing energy metabolism, supporting the therapeutic benefits of dapagliflozin. In the CLP and Sham groups, a negative correlation with arachidonic acid reflects the inflammatory suppression of Pdk1, which disrupts metabolic homeostasis and contributes to disease progression. Retreg1, a key regulator of autophagy and cellular stress responses, also exhibited correlations indicative of its dual roles. Between the CD and CLP groups, a positive correlation with Capryloylglycine suggests that dapagliflozin enhances autophagic flux and stress adaptation through Retreg1 activation, promoting cardioprotection. Conversely, in the CLP and Sham groups, a negative correlation with arachidonic acid reveals that inflammation suppresses Retreg1, impairing autophagic pathways and exacerbating disease pathology.

Furthermore, all selected differentially expressed genes and metabolites were mapped to the KEGG pathway database to identify shared metabolic and signaling pathways. Compared to the Sham group, the CLP group exhibited significant enrichment in pathways such as protein digestion and absorption, rheumatoid arthritis, and PI3K-Akt signaling, which collectively drive SIC progression through dysregulated protein metabolism, inflammatory responses, and disrupted cellular survival mechanisms. In contrast, the CD and CLP groups demonstrated significant modulation of pathways, including carbon metabolism, AMPK signaling, and beta-alanine metabolism, reflecting dapagliflozin’s ability to restore energy substrate utilization, promote mitochondrial bioenergetics, and enhance autophagic processes. These pathway alterations suggest that dapagliflozin addresses key metabolic and inflammatory disruptions, improving myocardial function and mitigating SIC progression. Together, these findings underscore the therapeutic potential of dapagliflozin in targeting the pathological mechanisms underlying SIC (Figure 6B).

## 4. Discussion

Dapagliflozin, widely recognized for its cardioprotective effects in chronic heart failure, has demonstrated anti-inflammatory, metabolic, and anti-fibrotic properties [32,33,34]. However, its role in SIC remains underexplored. This study provides robust evidence that dapagliflozin improves cardiac function and mitigates disease progression in SIC. Our clinical findings reveal that the pre-hospital use of dapagliflozin significantly reduces the incidence of MACE and improves survival outcomes. In a mouse model of SIC, dapagliflozin enhanced ventricular function, reduced pathological remodeling, and attenuated myocardial fibrosis. Multi-omics analysis further identified AMPK signaling and autophagy as pivotal pathways mediating these effects, uncovering key biomarkers with diagnostic and therapeutic potential.

While existing studies on SGLT2 inhibitors have primarily focused on chronic diseases such as heart failure, with numerous trials demonstrating their benefits in reducing mortality and improving cardiac outcomes [8,35,36,37], the potential of these drugs in acute settings, particularly in early-phase interventions, remains largely unexplored. Recent studies have shown that SGLT2 inhibitors can reduce the risk of septic shock in diabetic patients and mitigate lipopolysaccharide (LPS)-induced cardiac dysfunction in animal models by alleviating cardiac load and preventing ventricular remodeling [35,38,39,40,41,42]. These findings suggest that SGLT2 inhibitors may offer therapeutic benefits in sepsis-related conditions, especially in the early stages. Our research builds upon these observations but shifts the focus from managing symptoms to early intervention in SIC. Whereas prior studies have largely concentrated on later-stage symptom control and metabolic stabilization, our work emphasizes the importance of targeting early inflammatory cascades and metabolic dysregulation before irreversible cardiac damage occurs [43]. By intervening early in SIC, dapagliflozin offers a preventive strategy rather than a reactive one, potentially revolutionizing the management of SIC. Moreover, this study integrates transcriptomic and metabolomic analyses to systematically explore the molecular mechanisms underlying dapagliflozin’s cardioprotective effects. This comprehensive approach not only enhances our understanding of the drug’ s impact on metabolic pathways in sepsis but also provides valuable contributions to the broader body of research on SGLT2 inhibitors in acute settings.

Although dapagliflozin shows promising therapeutic potential in SIC and has a well-established safety profile in chronic conditions like diabetes and heart failure [44,45], its application in SIC requires careful consideration of timing, dosing, and safety due to the complex pathophysiology and clinical context of sepsis. Early intervention in the dynamic metabolic environment of sepsis is crucial for treatment success. Precise timing and optimal dosing are essential to maximize dapagliflozin’s protective effects on cardiac function while minimizing associated risks. Although current guidelines do not specify the exact timing for initiating SGLT2 inhibitors, McMurray et al. proposed a quadruple therapy sequence, positioning SGLT2 inhibitors as the first step in reducing mortality and hospitalization rates, which further supports the potential benefits of early SGLT2 inhibitor use, especially in patients with cardiovascular risk [46]. During the early stages of sepsis, when inflammation and metabolic dysregulation are most pronounced, initiating dapagliflozin therapy may help alleviate persistent inflammation, immune suppression, and catabolic syndrome (PICS), thereby reducing the long-term burden of myocardial fibrosis and heart failure [47]. Echocardiography plays a critical role in diagnosing SIC and assessing myocardial function; however, there is no single diagnostic parameter for SIC, nor are there specific electrocardiogram (ECG) findings that can directly diagnose it [48]. Biomarkers like cTnT and BNP are often elevated in patients with sepsis-induced heart failure, and integrating changes in these markers can guide clinicians in considering therapies like dapagliflozin [49]. The use of dapagliflozin should be tailored to each patient, taking into account cardiovascular risk, hemodynamic stability, and renal function. Based on heart failure guidelines, the optimal dosing for patients with cardiovascular risk, stable hemodynamics, and adequate renal function (eGFR ≥ 20 mL·min^−1^ [1.73 m^2^]^−1^) is 10 mg QD [50,51]. For patients with severely impaired renal function, cautious use and dose adjustments are necessary [52]. While dapagliflozin generally has a favorable safety profile, risks such as hypoglycemia and fluid imbalance in septic patients require ongoing glucose monitoring, hemodynamic assessment, and individualized treatment planning.

This study has several limitations that should be acknowledged. The retrospective cohort design may have introduced selection bias, and residual confounding cannot be entirely ruled out. Additionally, the study did not account for the use of other medications prior to and during hospitalization, which may have influenced patient outcomes. The absence of this information limits a comprehensive evaluation of the therapeutic landscape of SIC. Pharmacokinetic and pharmacodynamic variations in septic patients with fluctuating renal function were also not specifically investigated, leaving an important area for future exploration. Although the CLP mouse model successfully replicates the key pathological features of SIC, it may not fully capture the heterogeneity of sepsis in human patients. Despite these limitations, the study focused on evaluating the pre-hospital use of dapagliflozin and employed rigorous grouping and methodological designs to minimize bias. The findings remain robust and provide valuable preliminary evidence supporting the potential clinical application of dapagliflozin in the management of SIC.

To further optimize dapagliflozin’s application, this study suggests the following research directions: conduct large-scale, prospective, randomized controlled trials to comprehensively evaluate the efficacy and safety of dapagliflozin in various sepsis patient populations, with particular focus on the early treatment window and its potential impact on long-term cardiovascular outcomes; include detailed documentation of all medications used prior to and during hospitalization to assess the combined effects of multiple treatments on cardiovascular and overall outcomes, providing a more comprehensive understanding of dapagliflozin’s role in managing SIC; incorporate advanced molecular techniques, such as gene manipulation and pathway inhibition experiments, to confirm the molecular mechanisms underlying dapagliflozin’s effects and strengthen the biological foundation for its clinical application; and investigate pharmacokinetic and pharmacodynamic changes in septic patients with fluctuating renal function to elucidate how renal function variations influence the metabolism and clinical efficacy of dapagliflozin. By addressing these limitations and pursuing these research directions, future studies can refine the clinical application of dapagliflozin, ensuring its safety and efficacy in the management of SIC.

## 5. Conclusions

**Figure 7:**
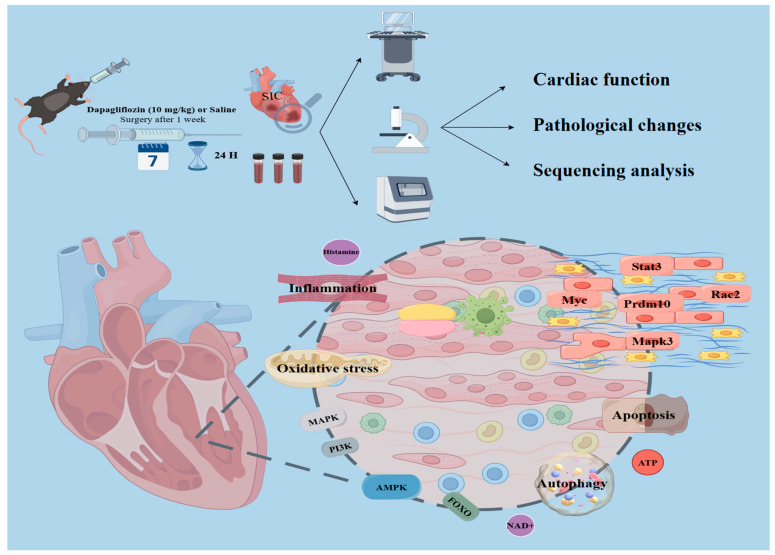
Schematic diagram of the protective mechanism of dapagliflozin in sepsis-induced cardiomyopathy: The SIC model was established by animal experiments. After one week of pretreatment with dapagliflozin (10 mg/kg), *mice* underwent CLP surgery to induce SIC, and cardiac function, pathological changes, and overall mechanism changes were evaluated 24 h after surgery. Treatment with dapagliflozin significantly improved cardiac function, reduced levels of myocardial injury markers, and attenuated histopathological damage in SIC *mice*. Mechanistically, dapagliflozin enhances autophagy, reduces oxidative stress and inflammatory response, and restores the balance of energy metabolism by regulating key genes (such as *Prdm10, Rac2, Myc, Stat3, Mapk3*) and activating the AMPK signaling pathway. Overall, dapagliflozin exerted cardioprotective effects through multiple regulations, ultimately improving myocardial function and reducing tissue damage in SIC *mice*.

This study demonstrates that pre-treatment with dapagliflozin significantly improves survival and reduces cardiovascular events in sepsis-induced cardiomyopathy (SIC) patients. These clinical findings are supported by our animal model, underscoring dapagliflozin’s potential as a therapeutic strategy for SIC. The cardioprotective effects of dapagliflozin are mediated through several mechanisms, including the regulation of inflammatory responses, immune modulation, promotion of autophagy, inhibition of apoptosis, and the restoration of energy metabolism balance (Figure 7). The combined action of these mechanisms positions dapagliflozin as a promising therapeutic option for sepsis-induced cardiomyopathy.

## Figures and Tables

**Table 1:** Baseline information for patients using dapagliflozin or not.

	DAPA(−)	DAPA(+)	t/Z	*p*
Age	70.303 ± 14.404 years	62.292 ± 17.399 years	1.899	0.063
MAP	79.330 (66.670, 92.670) mmHg	72.500 (64.885, 86.140) mmHg	−1.333	0.182
HR	89.000 (78.000, 109.000) bpm	94.500 (75.250, 119.250) bpm	−0.420	0.674
LVEF	47.000 (46.000, 48.000)%	48.000 (47.000, 49.000)%	−0.599	0.549
TnI	0.620 (0.540, 1.720) ng/mL	0.610 (0.500, 1.450) ng/mL	−1.819	0.069
CK-MB	5.600 (3.900, 7.900) ng/mL	4.100 (3.350, 5.900) ng/mL	−2.339	0.019 *
BNP	171.000 (131.000, 271.000) pg/mL	139.025 (106.877, 184.795) pg/mL	−2.093	0.036 *
Lac	2.000 (1.700, 2.400) mmol/L	2.000 (1.600, 2.000) mmol/L	−0.220	0.826
PaO_2_/FiO_2_	524.762 (500.476, 560.476)	522.857 (498.095, 543.120)	−0.542	0.588
IL-6	31.260 (15.430, 59.550) pg/mL	32.915 (19.365, 55.462) pg/mL	−0.008	0.994
PCT	2.160 (1.280, 4.240) ng/mL	2.295 (2.120, 4.682) ng/mL	−1.123	0.261
CRP	40.440 (20.030, 60.000) mg/L	33.520 (18.143, 52.165) mg/L	−0.614	0.539
APACHE	12.000 (10.000, 14.000)	10.000 (9.750, 12.000)	−1.998	0.046 *
SOFA	7.000 (6.000, 10.000)	4.500 (4.000, 5.250)	−4.509	<0.001 ***

Note: MAP: Mean Arterial Pressure, mmHg; HR: heart rate, beats per minute; LVEF: left ventricular ejection fraction, %; TnI: troponin I, ng/mL; CK-MB: Creatine Kinase-MB, ng/mL; BNP: B-type Natriuretic Peptide, pg/mL; Lac: lactate, mmol/L; PaO_2_/FiO_2_: PaO_2_/FiO_2_ Ratio, Ratio; IL-6: Interleukin-6, pg/mL; PCT: Procalcitonin, ng/mL; CRP: C-Reactive Protein, mg/L; APACHE II: Acute Physiology and Chronic Health Evaluation II score; SOFA: Sequential Organ Failure Assessment score. * Indicates statistically significant (*p* < 0.05). *** Indicates statistically significant *p* < 0.001.

## Data Availability

The original contributions presented in this study are included in the article. Further inquiries can be directed to the corresponding authors.
